# Influence of Amlodipine Enantiomers on Human Microsomal Cytochromes P450: Stereoselective Time-Dependent Inhibition of CYP3A Enzyme Activity

**DOI:** 10.3390/molecules22111879

**Published:** 2017-11-03

**Authors:** Kristyna Krasulova, Ondrej Holas, Pavel Anzenbacher

**Affiliations:** 1 Department of Pharmacology and Institute of Molecular and Translational Medicine, Faculty of Medicine, Palacky University at Olomouc, Hnevotinska 3, 775 15 Olomouc, Czech Republic; kkrasulova@seznam.cz; 2 Department of Pharmaceutical Technology, Faculty of Pharmacy in Hradec Kralove, Charles, Akademika Heyrovskeho 1203, 500 05 Hradec Kralove, Czech Republic; Holas.ondrej@gmail.com

**Keywords:** amlodipine, enantiomers, cytochrome P450, drug–drug interactions, enzyme inhibition, stereoselectivity

## Abstract

Amlodipine (AML) is available as a racemate, i.e., a mixture of *R*- and *S*-enantiomers. Its inhibitory potency towards nine cytochromes P450 (CYP) was studied to evaluate the drug–drug interactions between the enantiomers. Enzyme inhibition was evaluated using specific CYP substrates in human liver microsomes. With CYP3A, both enantiomers exhibited reversible and time-dependent inhibition. *S*-AML was a stronger reversible inhibitor of midazolam hydroxylation: the K_i_ values of *S*- and *R*-AML were 8.95 µM, 14.85 µM, respectively. Computational docking confirmed that the enantiomers interact differently with CYP3A: the binding free energy of *S*-AML in the active site was greater than that for *R*-AML (−7.6 vs. −6.7 kcal/mol). Conversely, *R*-AML exhibited more potent time-dependent inhibition of CYP3A activity (K_I_ 8.22 µM, K_inact_ 0.065 min^−1^) than *S*-AML (K_I_ 14.06 µM, K_inact_ 0.041 min^−1^). *R*-AML was also a significantly more potent inhibitor of CYP2C9 (K_i_ 12.11 µM/*S*-AML 21.45 µM) and CYP2C19 (K_i_ 5.97 µM/*S*-AML 7.22 μM. In conclusion, results indicate that clinical use of *S*-AML has an advantage not only because of greater pharmacological effect, but also because of fewer side effects and drug–drug interactions with cytochrome P450 substrates due to absence of *R*-AML.

## 1. Introduction

**Figure 1:**
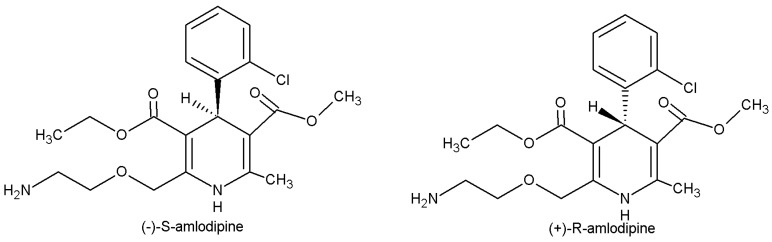
The structures of amlodipine enantiomers.

The dihydropyridine calcium channel antagonist amlodipine is one of the most commonly prescribed drugs for treatment of hypertension and angina [1,2,3]. It is commercially available as a racemic 1:1 mixture of *R*- and *S*-enantiomers (Figure 1). However, *S*-amlodipine is known to be 1000 times more pharmacologically potent than *R*-amlodipine and has therefore been marketed in enantiopure form under the name levamlodipine in some Asian countries [4,5,6]. Amlodipine is extensively (about 90%) converted to inactive metabolites via hepatic metabolism, with 60% of the metabolites being excreted in the urine together with 10% of the remaining parent compound. The drug is cleared via CYP3A-mediated dehydrogenation of its dihydropyridine moiety to a pyridine derivate, primarily yielding an inactive metabolite designated M9 [1,7]. 

Cytochrome P450 enzymes (CYPs) are important in the oxidative biotransformation of endogenous compounds and xenobiotics including drugs and environmental chemicals [8,9,10]. Drug–drug interactions that affect CYP activity can cause major problems in patients who are undergoing multi-drug treatment or who consume certain foods: the resulting enzyme inhibition can increase the risk of adverse reactions or reduce the effectiveness of prodrugs. Amlodipine reportedly reduces the activities of various cytochromes P450 including CYP1A1, CYP3A4, CYP2B6 and CYP2C9 [11,12]. The genetic polymorphism of CYPs may also significantly affect the efficacy and safety of some drugs, giving rise to the so-called “poor”, “intermediate”, “extensive” (also referred to as “normal”), and ultrarapid metabolism pharmacogenetic phenotypes [10]. This effect can be augmented by interactions between concomitantly taken drugs that are metabolized by the same enzymes. Many examples of genetic influences on the metabolism of clinically relevant drugs have been reported [13]. The CYP2C family accounts for approximately 28% of the total CYP protein content in human liver microsomes (HLM) [14], and CYP2C9 mediates the metabolism of 20% of all drugs that undergo phase I metabolism [15]. To date, at least 60 allelic variants of CYP2C9 and 35 allelic variants of CYP2C19 (www.pharmvar.org/) have been reported, many of which are associated with reduced substrate metabolism. Several studies have demonstrated the clinical significance of CYP2C9 and CYP2C19 polymorphisms [10]. 

It is reasonable to expect that the enantiomers of amlodipine may have different inhibitory effects on CYP-mediated drug metabolism. To investigate the inhibitory potency of *R*- and *S*-amlodipine, their IC_50_ and K_i_ values against nine relevant CYPs were assessed in HLM, and their selective effects were characterized. Because CYP2C9 and CYP2C19 are highly polymorphic and their polymorphisms are relevant to the pharmacodynamics and pharmacokinetics of many drugs, additional experiments were performed to characterize the amlodipine enantiomers’ inhibitory effects on CYP2C9 and CYP2C19 polymorphisms associated with extensive (normal), intermediate, and no enzyme activity. 

## 2. Results

### 2.1. Effects of Amlodipine Enantiomers on the Enzyme Activities of CYP in HLM

**Figure 2:**
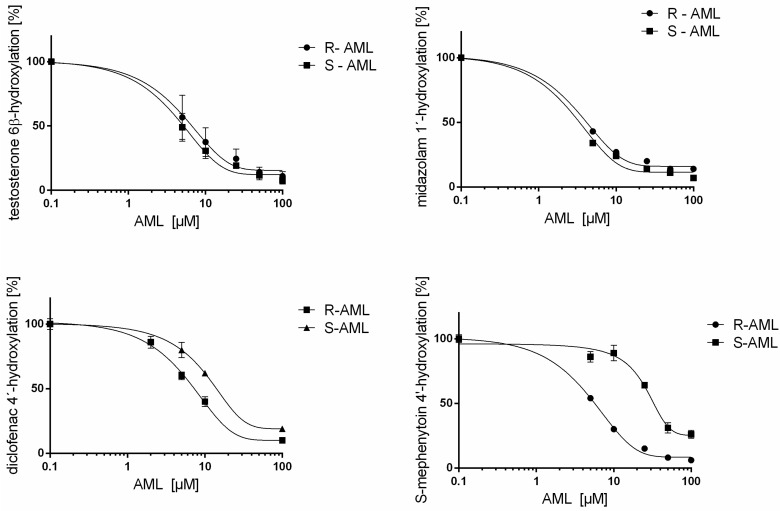
The effects of *R*- and *S*-AML on CYP3A-mediated midazolam 1′-hydroxylation, CYP3A-mediated testosterone 6β-hydroxylation, CYP2C9-mediated diclofenac 4′-hydroxylation and CYP2C19-mediated *S*-mephenytoin 4′-hydroxylation. Inhibition activities were determined as the means ± SD of two independent experiments performed in triplicate, and are expressed as the percentages of activity remaining relative to controls (i.e., the activity is 100% in the absence of the studied compounds). The activities of CYP3A, CYP2C9 and CYP2C19 were affected by both enantiomers and exhibited enantioselective inhibition.

Both *R*- and *S*-AML inhibited CYP3A (determined by evaluating their effects on testosterone 6β-hydroxylation and midazolam 1′-hydroxylation), CYP2B6 (7-ethoxy-4-(trifluoromethyl)coumarin 7-deethylation, EFCD), CYP2C9 (diclofenac 4′-hydroxylation), and CYP2C19 (*S*-mephenytoin 4′-hydroxylation). However, only *S*-AML inhibited the 6-hydroxylation of paclitaxel by CYP2C8. Additionally, neither AML enantiomer affected the activity of CYP1A (7-ethoxyresorufin *O*-deethylation), CYP2A6 (coumarin 7-hydroxylation), CYP2D6 (bufuralol 1′-hydroxylation), or CYP2E1 (chlorzoxazone 6-hydroxylation); in all these cases, the IC_50_ values for both AML enantiomers were above 50 μM (see Table 1 and Figure 2).

**Figure 3:**
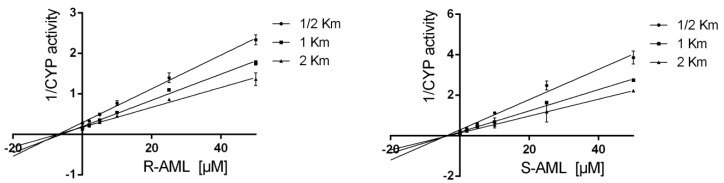
Dixon plots illustrating the noncompetitive inhibition of 6β-hydroxylation of testosterone (CYP3A) by *R*- and *S*-amlodipine after a 30-min preincubation. Each data point represents the mean ± SD of triplicate incubations with two independent measurements each.

Further experiments were performed with CYP3A, CYP2C9 and CYP2C19 to determine the mechanism of their inhibition by the AML enantiomers and the corresponding K_i_ values (Table 2). The inhibition of CYP3A was evaluated using preincubation times of 3 and 30 min, and two different probes (testosterone and midazolam). The inhibitors’ effects were evaluated by creating Dixon plots (Figure 3) as well as Lineweaver–Burk, and Scatchard plots (data not shown) of the enzyme activity as a function of the inhibitor concentration. Experiments with a three-minute preincubation period indicated that both enantiomers were competitive inhibitors, as would be expected given that AML is itself a substrate of CYP3A. However, there was significant enantiospecificity in the inhibition of midazolam hydroxylation: *S*-AML (*p* < 0.05, *N* = 8) was a stronger inhibitor than *R*-AML. Conversely, there was no detectable enantiospecificity in the inhibition of testosterone hydroxylation, see Table 2. 

Experiments with a 30-min preincubation period revealed an additional noncompetitive mechanism of inhibition (Figure 3). There were differences between the inhibitory potencies of *R*-AML and *S*-AML in the testosterone assay. However, the longer preincubation period eliminated the enantiospecificity of inhibition in the midazolam assay. Additionally, extending the preincubation period greatly extended the overall degree of CY3A inhibition in both assays (see Table 2). This behavior was suggestive of time-dependent inhibition, and was investigated further. 

Similar stereoselectivity was observed in studies on the inhibition of CYP2C9 and CYP2C19 (Table 2): *R*-AML inhibited the activity of CYP2C9 twice as strongly as *S*-AML (*p* < 0.05, *N* = 6). The Dixon, Lineweaver–Burk and Scatchard plots (data not shown) resulting from these assays fitted very well to the equations for the mixed model of inhibition. *R*-AML also inhibited CYP2C19 activity 12 times more strongly than *S*-AML (*p* < 0.005, *N* = 6). In this case, the Dixon, Lineweaver–Burk and Scatchard plots (data not shown) were fitted to the equation for noncompetitive inhibition.

### 2.2. Binding Pose of R- and S-AML in CYP3A4 Molecule

**Figure 4:**
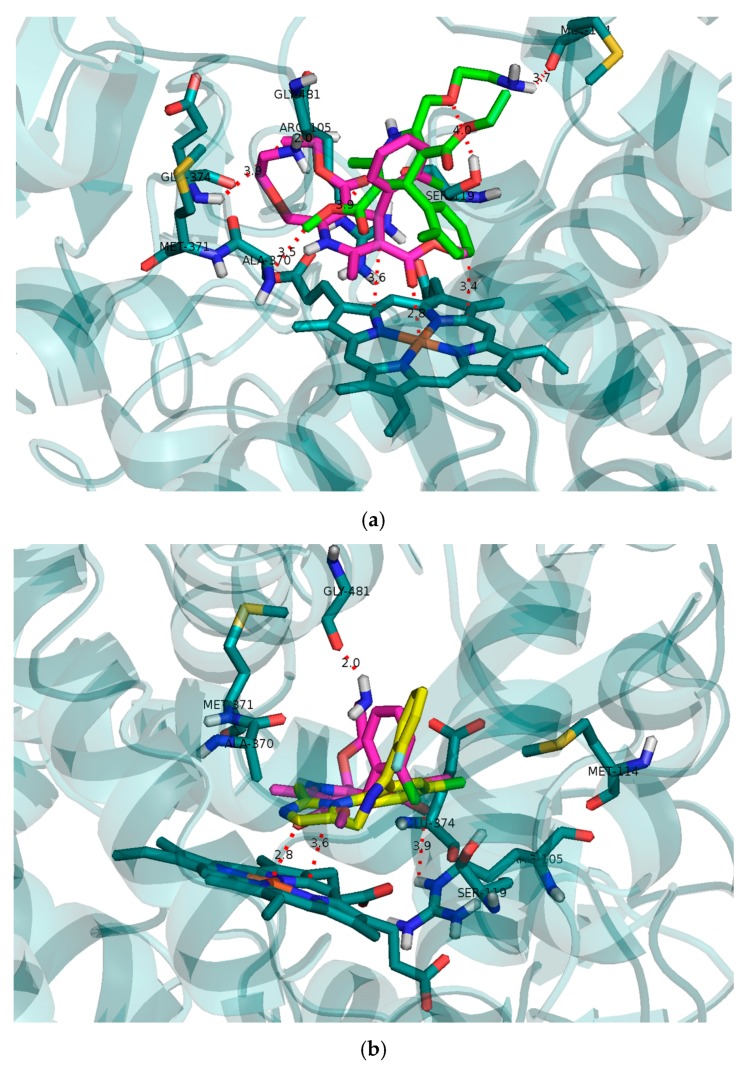
(**a**) Binding modes of *R*-AML (green) and *S*-AML (magenta) in CYP3A4; (**b**) The initial binding poses of midazolam (yellow) and *S*-AML (magenta). Receptor structure and residues are depicted in blue-grey.

Molecular modeling results reveal that there is indeed a distinct difference in interactions of *R*-AML and *S*-AML with CYP3A4 cavity (See Figure 4a). Both testosterone and midazolam assume position with the site of their oxidation with CYP3A4 by approaches the bottom of the cavity presented by heme. *S*-AML assumes this pose in greater proximity towards the cavity bottom and heme and thereby prevents more effectively heme to be approached by substrate molecules (Figure 4b). Extensive π–π stacking with heme is evident as well as direct interaction with heme iron and carbonyl oxygen of *S*-AML. The binding pose is stabilized by hydrogen bonds and Arg105 and Gly481. Such a pose presents plausible explanation of *S*-AML’s higher inhibitory activity. Nevertheless, *R*-AML is shown to be distinctly overlaid with the initial binding pose of midazolam, and facilitated relatively weaker interactions with heme via π–π interaction. The pose, if further stabilized by interactions with Met114, Ser119 and Ala307. *R*-AML’s inhibitory potential is not negligible as was shown by in vitro results. The free binding energy of −7.6 kcal/mol for *S*-AML also shows higher affinity towards the active site compared to the less potent *R*-isomer (−6.7 kcal/mol). This relatively firm binding of *S*-AML (the estimated free binding energy of −7.6 kcal/mol compared to −7.7 kcal/mol for midazolam) offers explanation for competitive inhibitory properties towards both midazolam and testosterone metabolism. The binding energy of testosterone being lower (−6.8 kcal/mol).

### 2.3. Time-Dependent Inhibition of CYP3A (Midazolam 1′-Hydroxylation) by Amlodipine Enantiomers

**Figure 5:**
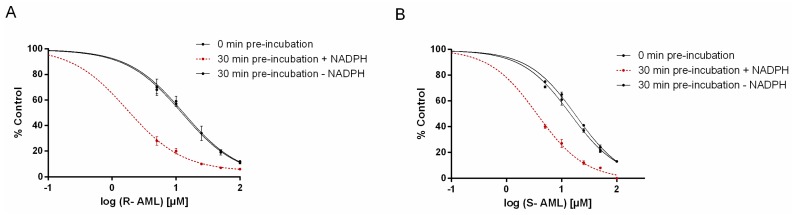
IC_50_ shift data for inhibition of 1′-hydroxymidazolam formation by the time-dependent inhibitors *R*- (**A**) and *S*-AML (**B**). *R*- and *S*-AML were preincubated at concentrations of 0–100 μM with HLM in the presence and absence of NADPH before being mixed with the CYP3A substrate, midazolam. Data are means ± SD for two separate experiments performed in triplicates. *R*- and *S*-AML are both reversible and time-dependent CYP3A inhibitors because they cause inhibition in both the absence and the presence of NADPH during the preincubation period.

**Figure 6:**
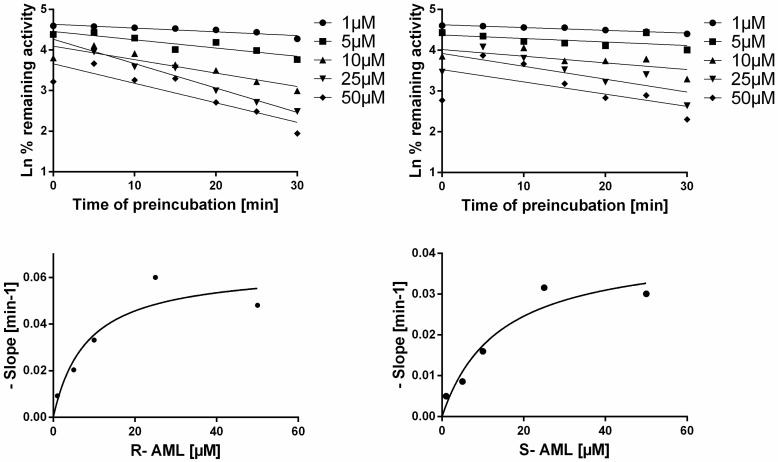
Upper panel shows inactivation plots for the time-dependent CYP3A inhibitors *R*- and *S*-AML based on the midazolam hydroxylation assay. Data represent means of duplicate incubations. Lower panel shows nonlinear regression plots of the negative slopes as functions of the inhibitor concentration, which were used to obtain K_inact_ and K_I_ values.

Time-dependent inhibition of enzyme activity is a characteristic feature of mechanism-based inhibition. The fact that the measured K_i_ values for *S*-AML and *R*-AML (Table 2) varied with the length of the preincubation period (3 or 30 min) suggested that these ligands are time-dependent CYP inhibitors. To test this hypothesis, *S*-AML and *R*-AML were preincubated with HLM (at a concentration 10-times higher than required in the assay) for 30 min with or without Dihydronicotinamide-adenine dinucleotide phosphate (NADPH), then diluted 10-fold and incubated with NADPH and midazolam. Preincubation with NADPH reduced midazolam hydroxylation by approximately by 55% (*R*-AML) or 45% (*S*-AML) relative to the case for preincubation without NADPH (results not shown), indicating that the NADPH facilitated the degradation of AML. The IC_50_ shift method was used to confirm that AML-mediated inhibition is both reversible and time-dependent [16]. Without preincubation, the IC_50_ values for *S*- and *R*-AML were 13.17 µM and 14.42 µM, respectively. A 30-min preincubation reduced these values to 1.73 µM for *S*-AML and 3.61 µM for *R*-AML. The presence of NADPH during the preincubation reduced the IC_50_ values for *S*- and *R*-AML 8-fold and 5-fold, respectively, confirming the time dependent nature of CYP3A inhibition by AML (Figure 5). Figure 6 shows the plots used to compute the inactivation rate constants for *R*- and *S*-AML. The corresponding estimated K_I_ and K_inact_ values are presented in the Table 3 together with K_I_/K_inact_ ratios indicating that *R*-AML is a stronger time-dependent inhibitor of the CYP3A enzymes.

### 2.4. Effects of Amlodipine Enantiomers on the Enzyme Activities of Various CYP2C9 and CYP2C19 Alleles

In addition to being a typical dihydropyridine substrate of CYP3A, AML inhibits other CYP enzymes such as CYP2C9 and CYP2C19, as demonstrated by the IC_50_ values presented in Table 3. CYP2C9 and CYP2C19 are two of the most important members of the CYP2C family, participating in the metabolism of the anticoagulant warfarin and antiplatelet thienopyridines such as clopidogrel. We therefore tested the drug–drug interactions of *R*- and *S*-AML in human liver microsomes genotyped for CYP2C9 and CYP2C19. The inhibition of characteristic CYP2C9 and CYP2C19 activities (diclofenac and *S*-mephenytoin hydroxylation) by AML was studied using genotyped microsomes corresponding to extensive enzyme activity (wild-type alleles), intermediate activity, and no (or poor) activity, as specified in Table 5. The IC_50_ values and inhibition rate parameters obtained for different alleles of the same CYP were then compared; these results are summarized in Table 4. The inhibition was stereoselective: *R*-AML was a stronger inhibitor than *S*-AML, in keeping with results obtained using pooled human liver microsomes. The IC_50_ values for *R*- and *S*-AML were 13.16 µM and 44.92 µM, respectively; against CYP2C9*1/*1; 12.98 µM and 53.19 µM, respectively; against CYP2C9*1/*2; 8.88 µM and 19.15 µM, respectively; against CYP2C19*1/*1; and 7.12 µM and 10.30 µM, respectively; against CYP2C19*1/*2. CYP2C9*1/*2 and CYP2C19*2/*2 exhibited reduced and no activity, respectively.

## 3. Discussion

Amlodipine is clinically available as a racemic mixture of *R*- and *S*-enantiomers, which are known to have different pharmacokinetic and pharmacodynamic properties. Compared to the *R*-enantiomer, the *S*-enantiomer exhibits approximately 1000-fold greater pharmacological activity, is eliminated more slowly, and has a longer t_1/2_ [17]. In addition, no racemization occurs in vivo when enantiopure AML is administered to humans [18]. In 2014, amlodipine was the sixth most commonly prescribed drug in the United States [19]. As a typical CYP3A substrate, it is known to influence the metabolism of several coadministered CYP3A substrates. A typical example is its effects on statins, with which it is commonly coprescribed in patients with hypertension and hypercholesterolemia: amlodipine can raise the level of simvastatin and increase the risk of myopathy. Consequently, the US Food and Drug administration has issued a recommended dose limitation for this drug combination [1,20]. The inhibition of CYP3A by amlodipine is also responsible for a diminished pharmacodynamic response to clopidogrel [21,22]. Also there was reported a significant in vivo interaction between amlodipine and tacrolimus resulting in rapid increase in tacrolimus blood concentrations [23]. The CYP3A4 is predominant hepatic form but also CYP3A5 contributes significantly to total liver CYP3A.Their substrate specificity overlaps and therefore it is referred here to these two enzymes together as CYP3A [24].

When dealing with racemic mixtures of compounds that inhibit CYP-mediated metabolism in vitro, it is important to consider the inhibitory potency of each enantiomer individually because several cases of stereoselectivity in CYP inhibition have been reported. For instance, *R*-omeprazole inhibited the 4-hydroxylation of diclofenac by CYP2C9 16 times more strongly than did *S*-omeprazole [25], the K_i_ value for (+)-ketoconazole towards CYP3A (6β-hydroxylation of testosterone) is five times lower than that for (−)-ketoconazole [26], the K_i_ value for *R*-tamsulosin towards CYP3A (6β-hydroxylation of testosterone) is five times lower than that for *S*-tamsulosin [27]. We have studied the inhibition of CYP3A enzymes extensively because these enzymes are responsible for most known drug biotransformation reactions, including the oxidation of amlodipine (which is mediated almost exclusively by CYP3A). Evidence for stereoselective inhibition of CYPs by some other dihydropyridine calcium channel blockers has been presented recently. For example, (+)-benidipine and (+)-felodipine were reported to strongly inhibit CYP3A and CYP2C19, respectively [28]. This work demonstrates that both *R*- and *S*-amlodipine are effective reversible and time-dependent inhibitors of CYP3A enzyme activities in human liver microsomes. *S*-AML is the stronger inhibitor if one only considers competitive inhibition (clearly indicated in shorter incubation times). However, time-dependent inhibition experiments showed *R*-AML to be the stronger inhibitor. Experiments with extended preincubation periods indicated that both mechanisms of inhibition become relevant in such cases (see Table 4). Amlodipine enantiomers act as competitive inhibitors and also exhibit the time-dependent inhibition. Time-dependent inhibition encompasses all phenomena that cause enzyme activity to decline as the incubation time increases. It can result from several different processes, but two are considered especially significant. One is “mechanism-based inhibition”, which typically involves the formation of reactive electrophiles that react with the CYP to form covalent adducts of the heme or apoprotein, inactivating the enzyme. The second involves the formation of tightly bound complexes between the P450 and specific metabolites (this process is also known as “quasi-irreversible” inhibition) [29,30]. Increased inhibitory potency (up to 8-fold) towards CYP3A activity when preincubated in the presence of NADPH suggest that AML may be converted, at least in part, to reactive intermediates or products that contribute to the overall inhibition of CYP3A activity. Time-dependent inhibition by racemic amlodipine was previously predicted by Jones et al. by computational approaches [31]. 

Molecular modelling of the initial binding poses for *R*- and *S*-AML in CYP3A4 was performed to compare their predicted capabilities as reversible competitive inhibitors to the results of the in vitro experiments. CYP3A4 has a relatively large and flexible active site cavity that can accommodate multiple substrate molecules to achieve optimal activity [32,33,34]. The modelling results confirmed the expected difference between the interactions of *R*- and *S*-AML with the CYP3A4 cavity (Figure 4a). Both testosterone and midazolam adopt binding positions that place their sites of oxidation by CYP3A4 in close proximity to the heme located at the bottom of the cavity. *S*-AML occupies a position closer to the heme than does *R*-AML, and is therefore more effective at preventing the approach of other substrate molecules. Together with the potential formation of π–π stacking interactions between the heme and the dihydropyridine ring of *S*-AML, this may explain *S*-AML’s greater inhibitory activity. The binding free energy of −7.6 kcal/mol for *S*-AML also reflects its higher affinity towards the active site compared to the *R*-isomer (−6.7 kcal/mol). 

Because *S*-AML is the pharmacologically active component of racemic amlodipine, the use of enantiopure *S*-AML could impose fewer burdens on patients and reduce the risk of drug–drug interactions. The absence of *R*-AML may be also beneficial in terms of cytochrome P450 inhibition. This suggestion was strongly confirmed by the finding that *R*-AML was a stronger inhibitor of both CYP2C9 and CYP2C19, having IC_50_ values against these enzymes that were two- and twelve-fold lower than those for *S*-AML, respectively (see Table 1). The differences between *R*- and *S*-AML in terms of their inhibition of CYP3A were minor and probably clinically insignificant, but treatment with enantiopure *S*-AML would halve the required amlodipine dosage required for effective treatment. If one also considers that *R*-AML causes venodilation and is responsible for side effects associated with racemic amlodipine, the use of the pure *S*-enantiomer becomes even more appealing [5]. Enantiopure *S*-AML is already used as a drug in India, China and some other Asian countries. Levamlodipine has a better safety profile than the racemate because it is administered at half the dosage and offers better tolerability with a reduced incidence of peripheral edema while retaining the antihypertensive effectiveness of conventional amlodipine [6]. 

Approximately 40% of human cytochrome P450 drug metabolism is carried out by polymorphic enzymes [35]. Extensive and intermediate metabolizers are more susceptible to drug interactions resulting from CYP inhibition than poor metabolizers. As discussed above, the pharmacokinetics of amlodipine in humans are stereoselective. In our inhibition studies, both enantiomers exhibited inhibition potential towards two polymorphic cytochromes P450: CYP2C9 and CYP2C19. We were interested in the possible differences between poor, intermediate and extensive metabolizer phenotypes of these two enzymes in terms of their stereoselective inhibition by *R*- and *S*-AML, and therefore investigated their responses to this drug using genotyped human liver microsomes (see Table 5). Kim et al. have previously evaluated the effects of different CYP3A5 genotypes on the disposition of the amlodipine enantiomers in humans, and found them to be minor [36]. This also seems to be true for CYP2C inhibition because we observed no significant differences in inhibition in extensive or intermediate metabolizers. The measured IC_50_ values indicate that the stereoselectivity of CYP2C inhibition was allele-independent: *R*-AML was the stronger inhibitor of CYP2C9*1/*1, CYP2C9*1/*2, CYP2C19*1/*1, and CYP2C19*1/*2. Naturally, the presence of loss-of-function variants in patient genome (CYP2C9 low and CYP2C19*2/*2) will be reflected in increase of plasma concentrations of the parent drug if the drug is a substrate of the CYP in question, which may lead to drug toxicity in some cases. These loss-of-function variants of CYP2C9 and CYP2C19 cannot hence be inhibited. 

Based on the above observations and the typical plasma concentrations (5 to 50 nM) of AML [17,37], its enantiomers can be regarded as weak to moderate inhibitors of CYP2C9 and CYP2C19 that exhibit stereoselective patterns only under special conditions (drug accumulation, overdosing, etc.). On the other hand, the inhibition of CYP3A by both enantiomers may affect CYP3A-mediated drug interactions in vivo. Our data are consistent with the results of a previous study that reported IC_50_ values for racemic amlodipine against CYP3A (5 µM), CYP2C9 (14 µM) and CYP2D6 (88 µM) [12]. However, this study provides new evidence on the mechanism of CYP3A inhibition, of reversible and of time-dependent CYP3A inhibition by amlodipine enantiomers. This work highlights the importance of accounting for stereochemistry when evaluating drug–drug interactions. We tested the influence of amlodipine enantiomers on the inhibition of important drug-metabolizing cytochromes P450 and showed that the enantiomers should be treated as pharmacologically independent entities. Our results showed for the first time that both amlodipine enantiomers are reversible (specifically, competitive) and time-dependent inhibitors of CYP3A activity, and inhibitors of CYP2C9 and CYP2C19. Differences in the inhibition potency of the AML enantiomers towards different metabolism phenotypes of CYP2C9 and CYP2C19 were also investigated, but found to be seemingly irrelevant. However, the individual AML enantiomers exhibited stereoselective inhibition of CYP3A and CYP2C.

## 4. Materials and Methods

*R*-amlodipine and *S*-amlodipine were purchased from Santa Cruz Biotechnology Inc. (Heidelberg, Germany). Ethoxyresorufin, 7-ethoxy-4-(trifluoromethyl)coumarin and 4′-hydroxydiclofenac were purchased from Fluka (Buchs, Switzerland). Coumarin, testosterone, diclofenac, bufuralol, chlorzoxazone, resorufin, 7-hydroxycoumarin, 7-hydroxy-4-(trifluoromethyl)coumarin, 1′-hydroxymidazolam and 1′-hydroxybufuralol were obtained from Sigma-Aldrich (Prague, Czech Republic). Midazolam was purchased from Abcam (Cambridge, UK), 6β-hydroxytestosterone was purchased from Ultrafine (Manchester, UK), and paclitaxel from Chemos CZ (Prague, Czech Republic). The 6-hydroxypaclitaxel and *S*-mephenytoin were purchased from Santa Cruz Biotechnology (Heidelberg, Germany), and (*S*)-4-hydroxy mephenytoin was bought from Toronto Research Chemicals Inc. (Toronto, Canada). Human liver microsomes and genotyped human liver microsomes (Table 5) were obtained from XenoTech (Lenexa, KS, USA). The activities of the CYP1A, CYP2A6, CYP2B6, CYP2C8, CYP2C9, CYP2C19, CYP2D6, CYP2E1, and CYP3A enzymes can be accessed from the XenoTech web site (www.xenotechllc.com). 

### 4.1. Enzyme Assays

The activities of the individual CYP forms are listed in Table 6 and were determined using established protocols (Table 6) and references therein [38]. The formation of metabolites from specific substrates was monitored using a Prominence HPLC system (Shimadzu, Kyoto, Japan) equipped with a LiChroCART 250-4 LiChrospher 100 RP-18 column or a Chromolith^®^ HighResolution RP-18 endcapped column (Merck, Darmstadt, Germany) and UV or fluorescence detection as specified in the relevant publications. Preliminary experiments were performed to determine the Michaelis constant (K_m_,) and limiting velocity (V_max_) of individual CYP forms. The incubation conditions used in each experiment were specific for the CYP form under consideration. In all cases, the conditions were chosen to be within the linear range for the reaction’s V_max_ in terms of time of incubation, substrate concentration (which was set equal to the enzyme’s K_m_), and the quantity of HLM in the reaction mixture (see Table 6). The reaction conditions used in the inhibition studies were identical to those used for the determination of individual CYP activities. The reaction mixtures were buffered with 100 mM K/PO_4_ (pH 7.4) or 50 mM K/PO_4_ (pH 7.4), and contained a NADPH-generating system consisting of isocitrate dehydrogenase, NADP+, isocitric acid, and MgSO_4_. Parallel methanol controls were performed for each experiment; in all such cases, the content of methanol in the reaction mixture was kept below 0.1% to exclude the possibility of enzyme inhibition at higher organic solvent (e.g., [39]).

The assays were performed using AML concentrations between 0 and 100 µM. Experiments were performed with the individual enantiomers alongside drug-free controls. Each incubation was performed in triplicate at 37 °C, and two independent measurements were performed for each replicate. Apparent K_i_ values were determined by performing additional measurements using substrate concentrations corresponding to 1/2 K_m_, K_m_, and 2 K_m_ in cases where inhibition was observed (i.e., where the tested drug achieved an IC_50_ < 10 μM). 

Inhibition of individual CYP activities was evaluated by plotting the remaining activity against the inhibitor concentration using GraphPad Prism (La Jolla, CA, USA). IC_50_ values were obtained by analyzing plots of the logarithm of the inhibitor concentration against the percentage of activity remaining after inhibition using the Sigma Plot 12 scientific graphing software (version 13.3.0, SPSS, Chicago, IL, USA). Apparent K_i_ values were determined by nonlinear regression analysis with GraphPad Prism, and are shown in Table 4. Prism was used to fit the experimental data to the equations presented by Copeland et al. [40]. The enzyme inhibition model (uncompetitive, mixed-model, competitive, or noncompetitive) applied to each data set was selected on the basis of visual inspection of Lineweaver–Burk, Dixon, and Scatchard plots, together with evaluations of model-derived parameters, *R*^2^ values, and absolute sum of squares values obtained from GraphPad Prism plots.

Stereoselective differences in the inhibitory effects of individual amlodipine enantiomers were analyzed using the Statistica 12 software package (12.1 StatSoft, Prague, Czech Republic). The Shapiro–Wilks test was used as a test of normality. The *t*-test was used for parametric data and the Mann–Whitney test for nonparametric data. 

### 4.2. The CYP3A Time-Dependent Inhibition

The single point assay was done as follows: *R*- or *S*-AML (25 μM) were preincubated with or without NADPH and HLM at a concentration of CYP 10-fold higher than required in the assay (125.6 pmol, to minimize potential reversible inhibition). After preincubation, an aliquot of the preincubation mixture was diluted 10-fold with buffer containing midazolam (at a final concentration corresponding to the K_m_) and incubated at 37 °C [41]. Subsequently IC_50_ values were determined under three different conditions: with 0-min of preincubation, with a 30-min preincubation in the absence of NADPH, and with a 30-min preincubation in the presence of NADPH to determined IC_50_ shift. The preincubation mixtures also contained HLM and the appropriate concentration of an AML enantiomer. Midazolam was added to the reaction mixture after the preincubation period, and the samples were then processed according to the standard inhibition protocol [42]. To find out the efficiency of inactivation, the K_I_ and K_inact_ values were assessed. The K_inact_ is the maximal rate of enzyme inactivation at a saturating concentration of the inhibitor, while the K_I_ is the inhibitor concentration that gives half the maximal rate of inactivation. The appropriate AML enantiomer at a concentration in the range of 0–100 μM was preincubated with HLM and NADPH for 0, 5, 10, 15, 20, 25, or 30 min, then an aliquot of the preincubation mixture was diluted with buffer containing midazolam (at a concentration equal to 5 times the K_m_) and NADPH. Experiments were also performed with methanol as a vehicle control. Each incubation was performed in duplicate at 37 °C, and two independent measurements were performed for enzyme inactivation at each enantiomer concentration. The natural logarithm of the corrected % remaining activity was then plotted against the preincubation time for each concentration of the inhibitor, and linear regression was performed using GraphPad Prism. The negative slopes obtained in this way (representing the observed initial rates of enzyme inactivation) were plotted against the inhibitor concentration, and the data were fitted by nonlinear regression (using GraphPad Prism) using the following equation: $\lambda =\frac{K_{inact}\cdot \left[I\right]}{K_{I}+\left[I\right]}$ where *λ* represents the rate constant for inactivation at each inhibitor concentration [*I*] [41,43].

### 4.3. Computational Docking to the CYP3A4 Active Site

Docking studies were carried out using the Molecular Operating Environment (MOE) software package (version 2015.1001; Chemical Computing Group Inc., Montreal, PQ, Canada) [40,41,43,44]. The CYP3A4 target structure was prepared from Protein Data Bank (PDB) structure 2V0M by removing the ligand and nonbonding water molecules and then subjecting the resulting structure to an energy minimization was performed (Gradient: 0.001 RMS kcal/mol/A2). Preliminary modeling results (data not shown) suggest that 2VOM geometry is the most favorable geometry to accommodate multiple ligands. The MOE site finder tool was used to identify the active site, the ligand structures were constructed using MOE’s incorporated ligand builder, hydrogens were added, partial charges assigned using the AMBER 94 forcefield, and energy was minimized to relieve strain within the protein structure. The compounds were then docked using the Alpha PMI placement algorithm (Samples per conformation: 30; No. of poses: 250). Rescoring was performed using the Affinity dG scoring function, then pose refinement was done using the induced fit method (Gradient: 0.001 kcal/mol; No. of iterations: 500, Cutoff distance 6 Å), and a second rescoring was performed using the GBVI/WSA dG scoring function. The Root-Mean-Square Deviation between the most highly scored crystallized inhibitor pose and the redocked 2V0M ligand was 1.05 Å.

## Figures and Tables

**Table 1:** Evaluation of amlodipine enantiomers as inhibitors of nine cytochromes P450 (CYPs) in human liver microsomes (HLM). The table shows IC_50_ values for both enantiomers against CYPs in HLMs. Bold numbers indicate cases where one enantiomer is a significantly stronger inhibitor than the other.

**IC_50_ (µM)**	**(CYP3A)**		**7-ethoxyresorufin (CYP1A2)**	**Coumarin (CYP2A6)**	**EFCD (CYP2B6)**
	**Testosterone**	**Midazolam**			
*R*-AML	3.43 ± 1.04	2.65 ± 0.05	47.39 ± 3.63	>50 µM	41.49 ± 1.37
*S*-AML	**1.99 ± 0.75**	2.18 ± 0.02	>50 µM	>50 µM	40.12 ± 1.11
**IC_50_ (µM)**	**Paclitaxel (CYP2C8)**	**Diclofenac (CYP2C9)**	***S*-mephenytoin (CYP2C19)**	**Bufuralol (CYP2D6)**	**Chlorzoxazone (CYP2E1)**
*R*-AML	>50 µM	**6.59 ± 1.17**	**5.14 ± 1.14**	>50 µM	>50 µM
*S*-AML	**9.55 ± 1.44**	16.42 ± 1.35	47.22 ± 1.91	>50 µM	>50 µM

**Table 2:** Mechanisms and strengths of inhibition of selected CYP activities by *R*- and *S*-AML. The table shows the K_i_ ± SD values of both amlodipine enantiomers towards various enzymes and the mechanism of inhibition in each case. Bold numbers indicate cases where one enantiomer is a significantly more potent inhibitor than the other.

CYP	*R*-AML		*S*-AML	
	K_i_ (µM)	Mechanism	K_i_ (µM)	Mechanism
Testosterone (CYP3A) ^a^	19.78 ± 3.91	competitive	16.32 ± 3.73	competitive
Midazolam (CYP3A) ^a^	14.85 ± 2.10	competitive	8.95 ± 1.48 *	competitive
Testosterone (CYP3A) ^b^	3.90 ± 0.55	noncompetitive	2.91 ± 0.36	noncompetitive
Midazolam (CYP3A) ^b^	3.86 ± 0.11	noncompetitive	3.24 ± 0.46	noncompetitive
Diclofenac (CYP2C9)	12.11 ± 1.53 *	mixed	21.45 ± 3.05	mixed
*S*-mephenytoin (CYP2C19)	5.97 ± 0.67 **	noncompetitive	72.2 ± 8.62	noncompetitive

^a^ 3 min preincubation time; ^b^ 30 min preincubation time. * *p* < 0.05; ** *p* < 0.005.

**Table 3:** The inactivation parameters, K_I_ and K_inact_, that characterize the time-dependent inhibition of CYP3A-mediated midazolam 1′-hydroxylation activity induced by *R*- and *S*-AML, K_inac_/K_I_ ratio determines the efficiency of inactivation.

	K_I_ (µM)	K_inact_ (min^−1^)	K_inac_/K_I_ (min^−1^·nM^−1^)
***R*-AML**	8.22 ± 2.78	0.065 ± 0.0069	7.91
***S*-AML**	14.06 ± 3.86	0.041 ± 0.0043	2.92

**Table 4:** IC_50_ values for *R*- and *S*-amlodipine characterizing their inhibition of genotyped CYP2C9 and CYP2C19 enzymes, as well as pooled human liver microsomes. Both phenotypes with poor and no activity reported no activity and the IC_50_ values were not measurable.

Enzyme	Allelic Variant	Metabolism Phenotype	IC_50_ (µM)	
			*R*-AML	*S*-AML
**Diclofenac (CYP2C9)**	CYP2C9*1/*1	extensive	13.16 ± 1.14	44.92 ± 5.09
	CYP2C9*1/*2	intermediate	12.98 ± 2.31	53.19 ± 6.94
	unspecified	poor	-	-
	pooled HLM	extensive	6.59 ± 1.17	16.42 ± 1.35
**S-mephenytoin (CYP2C19)**	CYP2C19*1/*1	extensive	8.88 ± 3.99	19.15 ± 2.52
	CYP2C19*1/*2	intermediate	7.12 ± 1.41	10.30 ± 2.55
	CYP2C19*2/*2	no	-	-
	pooled HLM	extensive	5.14 ± 1.14	47.22 ± 1.91

**Table 5:** Characterization of the genotyped microsomes: designation, genotype and phenotype of individual CYP2C9 and CYP2C19 enzymes.

Genotyped P450 Enzyme	Genotype	Metabolism Phenotype
H2C9.HA	CYP2C9*1/*1	extensive activity
H2C9.MA	CYP2C9*1/*2	intermediate activity
CYP2C9 low	unspecified	poor activity
H2C19.HA	CYP2C19*1/*1	extensive activity
H2C19.MA	CYP2C19*1/*2	intermediate activity
H2C19.NA	CYP2C19*2/*2	no activity

**Table 6:** Reactions used to evaluate the activity of different CYP forms and key experimental conditions used in each case. The substrate concentrations were set to the K_m_ for the enzyme/substrate combination, specified substrate, and the quantities of HLM used are expressed in terms of the amount of the CYP enzyme present in the reaction mixture.

P450 Enzyme	Specific Reaction	Substrate Concentration (µM)	HLM (nM)	Reaction Volume (µL)
CYP1A	7-ethoxyresorufin *O*-deethylation	1.4	350	100
CYP2A6	coumarin 7-hydroxylation	14.3	350	100
CYP2B6	7-ethoxy-4-(trifluoromethyl)coumarin 7-deethylation	15.3	350	100
CYP2C8	paclitaxel 6-hydroxylation	45.1	350	200
CYP2C9	diclofenac 4′-hydroxylation	20.7	175	200
CYP2C19	*S*-mephenytoin 4′-hydroxylation	28.2	250	200
CYP2D6	bufuralol 1′-hydroxylation	56.4	350	200
CYP2E1	chlorzoxazone 6-hydroxylation	56.4	160	1000
CYP3A	testosterone 6β-hydroxylation	97.2	200	500
	midazolam 1′-hydroxylation	2.2	1256	100

## Abbreviations

AML	Amlodipine
EFCD	7-Ethoxy-4-(trifluoromethyl)coumarin
HLM	Human liver microsomes
MOE	Molecular Operating Environment
NADPH	Dihydronicotinamide-adenine dinucleotide phosphate
PDB	Protein Bank Database
